# novoStoic2.0: An integrated framework for pathway synthesis, thermodynamic evaluation, and enzyme selection

**DOI:** 10.1371/journal.pcbi.1012516

**Published:** 2025-08-06

**Authors:** Vikas Upadhyay, Mohit Anand, Costas D. Maranas

**Affiliations:** Department of Chemical Engineering, The Pennsylvania State University, University Park, Pennsylvania, United States of America; CSIR-National Chemical Laboratory: CSIR National Chemical Laboratory, INDIA

## Abstract

Computational pathway design and retro-biosynthetic approaches can facilitate the development of innovative biochemical production routes, biodegradation strategies, and the funneling of multiple precursors into a single bioproduct. However, effective pathway design necessitates a comprehensive understanding of biochemistries, enzyme activities, and thermodynamic feasibility. Herein, we introduce novoStoic2.0, an integrated platform that combines tools for estimating overall stoichiometry, designing de novo synthesis pathways, assessing thermodynamic feasibility, and selecting enzymes. novoStoic2.0 offers a unified web-based interface as a part of the AlphaSynthesis platform (http://novostoic.platform.moleculemaker.org/) tailored for the synthesis of thermodynamically viable pathways as well as the selection of enzymes for re-engineering required for novel reaction steps. We exemplify the utility of the platform to identify novel pathways for hydroxytyrosol synthesis, which are shorter than the known pathways and require reduced cofactor usage. In summary, novoStoic2.0 aims to streamline the process of pathway design contributing to the development of sustainable biotechnological solutions.

## Author summary

Designing biosynthetic pathways for novel chemical targets often involves a series of non-trivial tasks: estimating stoichiometries, assessing thermodynamic feasibility, and selecting appropriate enzymes, typically addressed using separate tools, which can lead to inconsistencies and hinder the transition from computational design to experimental implementation. To address this, we developed novoStoic2.0, an integrated platform that unifies these tasks into a single workflow. It supports the construction of biosynthetic routes that are thermodynamically viable, meaning they are energetically favorable and chemically feasible, and helps identify which steps may require enzyme re-engineering. This streamlines the transition from computational design to experimental implementation. We demonstrated the platform’s capabilities by designing biosynthetic pathways for hydroxytyrosol, a compound with both industrial and biomedical relevance as an antioxidant. The resulting routes are shorter than known alternatives and reduce cofactor requirements, offering more efficient options for microbial production. By combining pathway construction, thermodynamic analysis, and enzyme selection in a coherent system, novoStoic2.0 simplifies and strengthens early-stage biosynthesis planning. We see this as a step toward faster, more reliable development of sustainable production routes in synthetic biology and metabolic engineering.

## Introduction

Advances in synthetic biology offer considerable potential for engineering biochemical pathways in producing a diverse array of molecules, ranging from biofuels and pharmaceuticals to value-added chemicals and environmentally friendly biodegradation strategies [1–6]. Traditional approaches to biosynthesis often rely on assembling cataloged enzymatic activities, limiting the exploration of novel production routes [7]. Recent studies have demonstrated the utility of leveraging enzyme promiscuity, wherein enzymes exhibit activity on substrates beyond their native targets [8], to enable the assembly of novel pathways through alteration of enzymatic substrate or cofactor specificity. However, the search for novel conversions through enzyme modification presents both a significant enzyme engineering challenge but also an opportunity [9–11]. In an example of a successful application the promiscuous hydroxylase enzyme (4-hydroxyphenylacetate 3-monooxygenase) was used to alter substrate specificity from its native substrate 4-hydroxyphenylacetate to tyrosol and tyramine. [12] These alternative pathways not only minimized the cell metabolic burden by lowering protein synthesis costs but also improved the efficiency of hydroxytyrosol production by rearranging the metabolic flux.

Several pathway design tools are available to generate sequential reaction steps to convert a source chemical into a target molecule. Notable examples include novoStoic [7], RetroPath 2.0 [13,14], and BNICE [15], which facilitate the exploration of biochemical pathways. User-friendly web interfaces are provided by tools such as RetroBioCat and novoPathFinder [16,17]. Moreover, recent advancements in machine learning ushered transformer-based models to move beyond the simple molecular-input line-entry system (SMILES) to a Seq2Seq model [18] akin to a large-language model (LLM) [19]. In addition, sampling techniques such as Monte Carlo tree search (MCTS) [18,20] or deep learning-guided AND-OR tree search [20–22] can be used to explore the identification of routes connecting the target molecules with inexpensive precursors.

Although pathway synthesis tools can invoke novel steps to complete biosynthetic pathways, the discovery or redesign of an enzyme to carry out the hypothesized transformation remains a challenge [23–25]. Recently, we developed EnzRank [23], which relies on utilizing convolutional neural networks (CNNs) to understand underlying residue patterns and combines with the substrate molecule signature to provide a probability score for the compatibility of enzyme-substrate pairs. However, implementing the novel steps requires *de novo* enzyme design or protein re-engineering to alter substrate specificity using directed evolution. One such example is the design of a luciferase enzyme that selectively catalyzes the oxidative chemiluminescence of novel luciferin substrates, diphenylterazine and 2-deoxycoelenterazine [26].

Thermodynamic feasibility assessment of the entire pathway and also of individual steps is an important check, as most databases used to train ML retrosynthesis tools, treat reactions as reversible, resulting in erroneously adding steps in a thermodynamically unfavorable direction. Tools such as eQuilibrator [27] and dGPredictor [28] can estimate the standard Gibbs energy change of reactions. While eQuilibrator uses an expert-defined functional group, dGPredictor uses automated chemical moieties that classify every atom in a molecule based on their surrounding atoms and bonds to feature molecules. Using structure-agnostic chemical moieties in dGPredictor allows for the estimation of the standard Gibbs energy of reactions containing novel metabolites absent from databases or molecular structures that cannot be decomposed using expert-defined functional groups.

**Fig 1:**
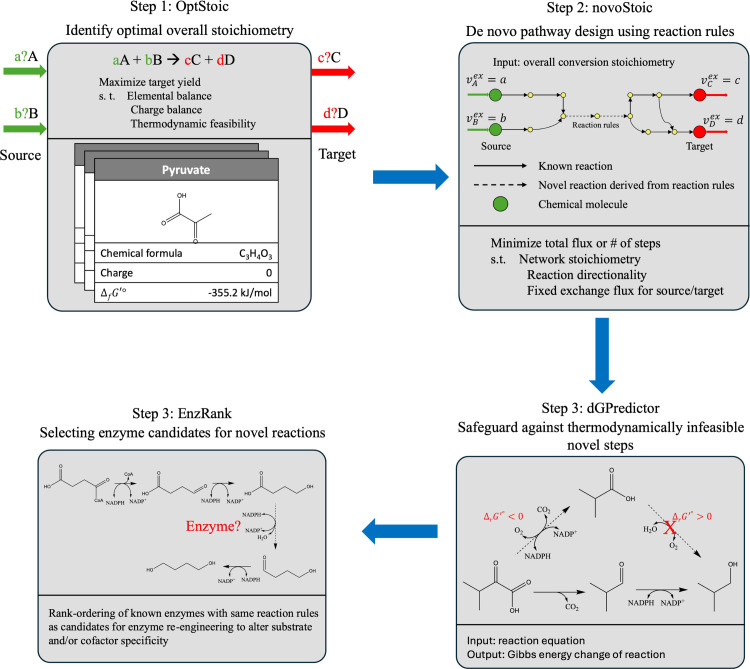
novoStoic2.0: overall workflow for the integrated pathway design, including tools for optimal overall stoichiometry of conversion, optStoic. De novo pathway design using optimal stoichiometry, novoStoic. Thermodynamic assessment of reaction steps using dGPredictor and rank-ordering of known enzymes for novel substrate activity to identify starting enzymes for novel steps using EnzRank.

Herein, we introduce the novoStoic2.0 framework, which integrates the aforementioned tasks (Fig 1) within a single interface. First, the optStoic [29] tool can be used to estimate the optimal overall stoichiometry of the desired conversion by maximizing the yield of the target molecule from the given starting compound. Next, novoStoic [7] attempts to identify the link between the input and output molecules of the overall conversion using both the database and novel reactions. To assess the thermodynamic feasibility of these individual steps, including novel ones, dGPredictor [28] is accessed to estimate the standard Gibbs energy changes. Finally, EnzRank [9] can be used to select enzyme candidates for (any) novel conversions identified within the pathway design.

novoStoic2.0 is a one-stop web-based interface for designing biosynthesis pathways that are not only thermodynamically feasible and carbon/energy balanced but also provide suggestions on enzyme selection for re-engineering associated with novel reaction steps.

### Design and implementation

This section describes the design, implementation, and use of the novoStoic2.0 user interface. We used a Streamlit-based Python framework to build the user interface for all the tools that are integrated within novoStoic2.0. We used the MetaNetX database to extract a total of 74,612 reactions and 1,292,153 molecules. Upon processing the database by removing unbalanced reactions, transport reactions, and reactions containing generic molecules, we have 23,585 reactions. Similarly, for molecules, we removed the generic molecules, molecules with multiple MetaNetX IDs, and used molecules that were present in 23,585 reactions, which ended up using 17,154 molecules from the database. Hence, in novoStoic2.0, we use 23,585 reactions and 17,154 molecules for pathway design. Using that, we generated the molecular signature for the molecules and reaction rules for the reactions that are used in novoStoic to design de novo pathways for biosynthesis. Finally, we generated 9,686 unique reaction rules from 23,585 reactions. However, both dGPredictor and EnzRank use the KEGG database for standard Gibbs energy estimations and enzyme selection, respectively. We created a mapping of MetaNetX IDs with KEGG IDs, in order to use the novoStoic outputs in both tools. If a MetaNetX ID is not present in KEGG, we use InChI and SMILES string and consider them as novel molecules in dGPredictor and EnzRank. Using dGPredictor to estimate the standard Gibbs energy change for novoStoic identified reactions, we generate thermodynamically feasible pathways. Allowing the integration with EnzRank, we probe all the enzyme sequences for a given reaction rule from the KEGG and Rhea databases using the KEGG REST API [30]] and Rhea API [31] along with the novel substrate SMILEs string to estimate the probability score for enzyme-substrate activity and rank-order the known enzymes for the novel reaction steps.

## Results

### novoStoic2.0 user interface

**Fig 2:**
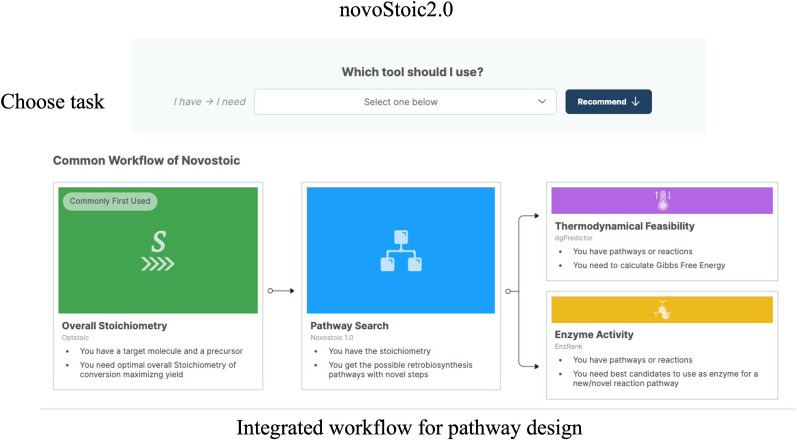
Annotated workflow of the home page of the novoStoic2.0 user interface. This page provides an option for users to select specific tasks, standalone as well as in the given workflow, to include all the tools for designing pathways.

Fig 2 illustrates the homepage of the web-based platform built using the Streamlit Python package.

**Fig 3:**
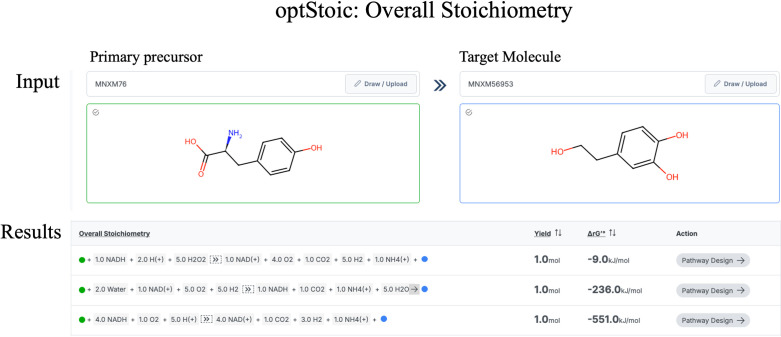
User interface of optStoic, requires the molecule information (MetaNetX/KEGG ID or SMILES string) of the starting and target molecules and outputs the optimal overall conversion stoichiometry, i.e., used in novoStoic to find synthesis pathways.

It provides brief information for each tool along with relevant references. optStoic [29] uses MetaNetX [30,32] as well as KEGG database compound IDs to input both the source and target molecules. Users may also specify any other co-substrate and co-product to be considered in conjunction with the source and target molecules. Subsequently, optStoic (interface shown in Fig 3) solves an LP optimization problem, generally aiming to maximize theoretical yield while ensuring mass, energy, charge, and atom balance [29]. The resultant output of this step is the overall target stoichiometry of the conversion.

**Fig 4:**
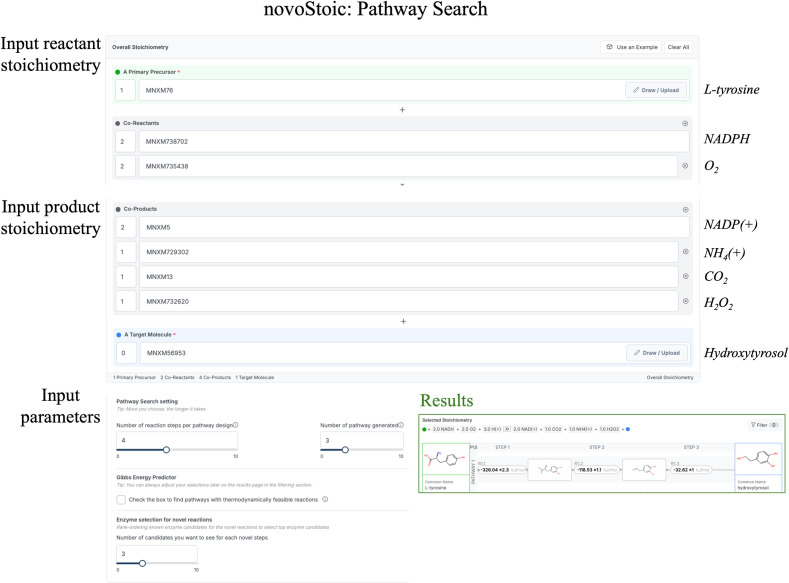
User interface of novoStoic, requires overall balanced stoichiometry of conversion from a source to a target molecule, and input parameters such as maximum number of steps for pathway search, number of pathways to search, and the number of top known enzyme candidates ranked by EnzRank for novel steps identified in the pathways.

This overall stoichiometry becomes a key input for novoStoic, depicted in Fig 4. novoStoic requires additional inputs, including the maximum number of (novel) steps, the maximum number of pathway designs, and the primary source and target molecules.

The output of novoStoic visualizes the identified pathways, as well as individual reactions with their standard Gibbs energy estimated using dGPredictor [28]. For pathways involving novel steps, the output provides the option of using EnzRank to generate the top known enzyme candidates for potential activity with a novel substrate (i.e., the default is the top five enzyme candidates for each novel step).

**Fig 5:**
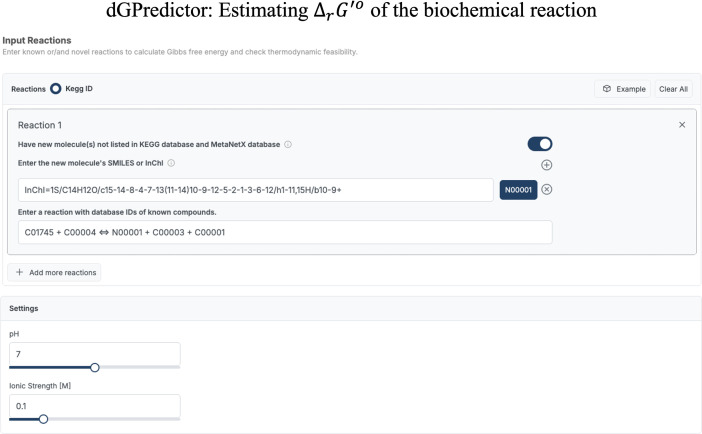
User interface of dGPredictor, requires the KEGG reaction string as input. An InChI string is required if a molecule is not present in the database. The user can modify settings such as pH and ionic strength to get $\Delta_{r}G^{\prime o}$ at different physiological conditions.

dGPredictor could also be used as a standalone standard Gibbs energy estimator as illustrated in Fig 5 using either the KEGG [30] reaction string or the InChI string for novel molecules that are not present in the KEGG [30] database. This feature holds broad applicability, including conducting global thermodynamic feasibility analysis of genome-scale metabolic models [15].

**Fig 6:**
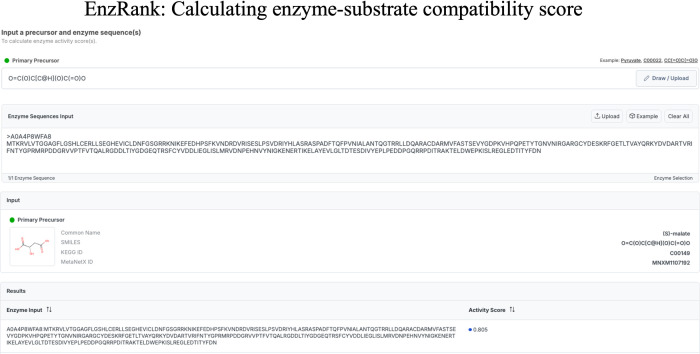
User interface for EnzRank, requires the SMILES string of the substrate and the enzyme sequence as input to estimate the probability score for the enzyme-substrate compatibility.

Finally, as shown in Fig 6, we also provide the user interface of EnzRank as a standalone tool within novoStoic2.0. Here, the users can input the enzyme’s amino acid sequence and the KEGG ID or SMILES string (if there is a novel structure) of the substrate to generate the probability score for the enzyme-substrate activity. This score can rank-order known enzymes for novel substrate activity. While the tool automatically uses enzyme sequences from KEGG and RHEA databases [30,31], users also have the option to manually input custom enzyme sequences if they are working with enzymes not included in these databases, offering greater flexibility in enzyme selection. EnzRank is also integrated during pathway search to extract and rank-order appropriate enzyme candidates for any novel steps found.

### Example application: Hydroxytyrosol synthesis

Hydroxytyrosol (HXT) is a powerful antioxidant that offers protection from free radicals. The initial effort of biosynthesis uses tyrosine as a starting substrate, using tyrosol as the intermediate, and was reported to achieve a yield close to 50% [12]. A recent effort by Chen et. al [12] uses a promiscuous hydroxylase enzyme to identify two novel pathways for HXT biosynthesis. Motivated by these results herein, we applied novoStoic2.0 to explore additional pathway designs. We show two of the identified new pathways designed using L-tyrosine as a starting precursor and HXT as the target molecule.

The first step involves the identification of the optimal stoichiometry (i.e., max carbon yield) for the overall conversion using optStoic. We explored multiple alternatives in one run by allowing as reactants/products an inclusive set of small molecules such as, CO_2_, H_2_O, NH_4_^+^, O_2_, H_2_O_2,_ and cofactors such as NAD(P)H, NAD(P)^+^ to balance the overall conversion.

optStoic identified the overall stoichiometry associated with the pathway identified by Chen et. al that uses a promiscuous hydroxylase enzyme given by:

**Overall Stoichiometry 1: Known pathway**


$$C_{9}H_{11}NO_{3}+2NAD(P)H+3H^{+}+2O_{2}\underset{\to }{-484.28\pm 1.42\frac{kJ}{mol}}C_{8}H_{10}O_{3}+NH_{4}^{+}+CO_{2}+H_{2}O_{2}+2NAD(P{)}^{+}$$ (1)

     L-tyrosine                    HXT

However, optStoic also identified the simpler 1 Tyr ◊ 1 HXT overall stoichiometry with the fewest co-products and co-substrates, and no cofactor usage:

**Overall Stoichiometry 2**


$$C_{9}H_{11}NO_{3}+H^{+}+H_{2}O_{2}\underset{\to }{-219.65\pm 1.41\frac{kJ}{mol}}C_{8}H_{10}O_{3}+NH_{4}^{+}+CO_{2}$$ (2)

            L-tyrosine              HXT

As shown above, both stoichiometries involve a high negative overall Gibbs energy change, alluding to their thermodynamic feasibility.

**Fig 7:**
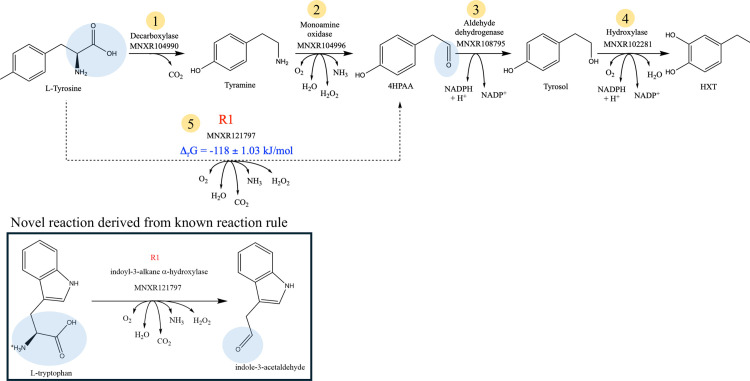
Pathway identified using novoStoic with overall stoichiometry 1, here is the pathway with steps 1-4 showing the known pathway from the literature. Whereas the pathway with steps 5-3-4 shows a novel pathway that bypasses steps 1 and 2 in a single step. Here, step 1 is a decarboxylation reaction, step 2 is tyramine: oxygen oxidoreductase, step 3 is aldehyde dehydrogenase, step 4 is a hydroxylase reaction, and novel step 5 comes from a reaction MNXR121797 (exact chemical transformation highlighted in blue), which is a hydroxylase enzyme.

The next step was to input these identified stoichiometries in novoStoic to construct complete pathways. Using stoichiometry 1, novoStoic found multiple pathways that have not been explored to our knowledge (see S1 File). Fig 7 illustrates these pathway alternatives, which include the existing pathway from the literature [12] alongside an alternate route.

The known pathway involves steps 1–4 (Fig 7) converts L-tyrosine to tyramine via tyrosine decarboxylase enzyme (EC 4.1.1.25) followed by the hydroxyl group addition at the meta-position to convert tyramine to 4HPAA using tyramine: oxygen oxidoreductase, followed by dehydrogenase and hydroxylase enzymes to convert 4HPAA to tyrosol and HXT, respectively.

However, an alternative novel pathway was identified that bypasses the first two steps of converting tyrosine to 4HPAA by integrating them into a single step using the reaction rule R1 (i.e., EC 1.13.99.-) derived from reaction indoyl-3-alkane-α-hydroxylase. The pathway in Fig 7 shows the novel bypassing step 5, which follows the reaction rule of reaction MNXR121797. Using dGPredictor, we confirmed that the proposed novel step involves a negative standard Gibbs energy. Reaction MNXR121797 (EC 1.13.99.-) has a single enzyme sequence assigned to it, which can serve as the starting enzyme that would need to be re-engineered for usher activity on the new substrate L-tyrosine. This makes the use of EnzRank redundant. Even though the reaction for step 4 is not cataloged in MetaNetX, it follows the reaction rule of reaction MNXR102281. However, recent experimental evidence [12] points out that enzyme 4-hydroxyphenylacetate 3-monooxygenase has a secondary activity on tyrosol, obviating the need for enzyme discovery/engineering.

**Fig 8:**
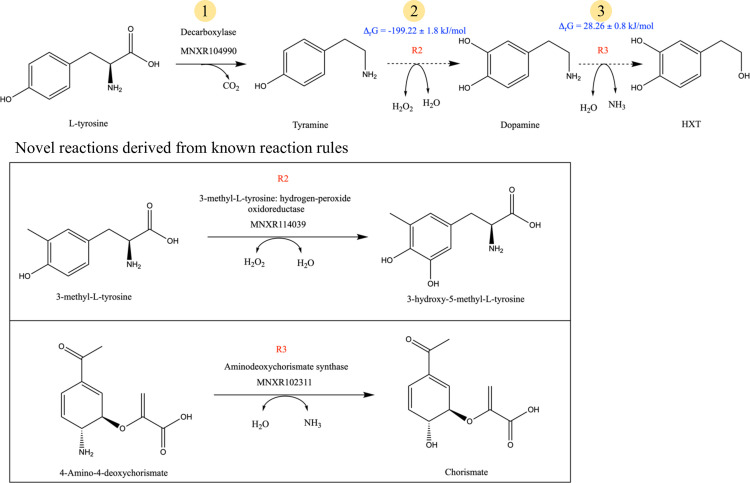
Pathway identified using novoStoic with overall stoichiometry 2, here is the pathway with steps 1-3 showing the shorter novel pathway. Here, step 1 is a known decarboxylation reaction, step 2 is derived from the reaction rule R2, and step 3 is derived from reaction rule R3.

Using optimal stoichiometry 2, we identified novel pathways using three steps that do not use any cofactors and only small molecules, co-reactants and co-products (see S1 File). Fig 8 shows one of the pathways identified by novoStoic for the synthesis of HXT.

Fig 8 illustrates the three-step cofactor-free pathway. Step 1 is the same as in the known pathway shown in (Fig 7) [12]. The novel reaction associated with step 2 follows the reaction rule associated with reaction 3-methyl-L-tyrosine hydrogen peroxide oxidoreductase (EC 1.11.2.5) that performs a hydroxyl addition to convert tyramine to dopamine. Notably, there is a known reaction in the MetaNetX database that performs this conversion, but it requires NAD(P)H as a cofactor. Using dGPredictor, we assessed the thermodynamic feasibility of the desired direction and found a high negative standard Gibbs energy. Step 3 is a novel reaction that shares the same reaction rule as reaction MNXR102311 (aminodeoxychorismate synthase), which converts 4-amino-4-deoxychorismate to Chorismate. dGPredictor assesses the standard Gibbs energy change to be slightly positive, which can be made feasible by tilting the reactant concentration to move the reaction in the forward direction. Nevertheless, this example showcases that novoStoic can identify a shorter pathway with a lesser number of cofactors compared to the known pathway. The novel step R2 (Fig 8) was already suggested in the recent article by Chen et. al [12] that includes the engineering promiscuous enzyme 4-hydroxyphenylacetate 3-monooxygenase to show activity with tyramine. However, novoStoic qualified it as a novel step as the reaction was not present in the database yet. For the novel step R3 (in Fig 8), we found a total of 3,744 unique enzyme sequences from the reactions with the same rule as R3 (see S2 File, which provides the list of all the enzymes for step R3). Next, EnzRank was used to rank-order known enzymes for the same reaction rule from which the novel step R3 is derived using the probability score to identify the potential of an enzyme to act on a substrate (dopamine). Here, Table 1 shows the top 3 candidates found using EnzRank for reaction step R3, and S3 File contains the EnzRank scores for all the enzyme sequences for the same rule as R3.

**Table 1:** Top 3 genes from different organisms selected by EnzRank for novel reaction step R3 with reaction rule derived from aminodeoxychorismate synthase enzyme.

Organisms	EnzRank score
*Streptomyces rochei*	0.87
*Streptomyces griseofuscus*	0.86
*Cellulomonas xiejunii*	0.80

Using HXT as an example/case study, we highlighted how novoStoic2.0 can be used to find multiple pathway alternatives for HXT synthesis mapping to different overall stoichiometries. These alternatives mapping to different overall stoichiometries were subsequently subjected to thermodynamic feasibility analysis, and enzymes were prioritized for all novel steps. Additional examples are provided in the S4 File.

In collaboration with the Molecule Maker Lab Institute (MMLI) at UIUC, novoStoic2.0 is being developed as an interactive website for the synthesis planning tool, which is a part of the AlphaSynthesis platform and is publicly available at http://novostoic.platform.moleculemaker.org/.

### Availability and future directions

Pathway design entails multiple tasks ranging from decisions on starting points and possible co-reactants/products, sequence of metabolic reaction steps, including novel reaction by-passes, selection of enzymes for uncharacterized reaction steps, checking on thermodynamic feasibility of chosen reaction direction, and many more. novoStoic2.0 integrates many of these steps within a single resource, thus streamlining the task of pathway discovery and evaluation. In contrast to existing pathway design tools, which focus solely on pathway generation and require separate analyses for thermodynamics, enzyme selection for individual steps, and overall reaction balance including cofactors, novoStoic2.0 integrates all these aspects into a unified framework. Table 2 presents a comparative analysis of the features offered by existing biosynthetic pathway design tools and novoStoic2.0. By enabling the rapid computational exploration of design alternatives, it expands the space of alternatives explored and thus the chances of success. The development of an easy-to-use web-based interface consolidates many tasks into a single platform. The web platform is currently limited to two runs due to computational constraints, and jobs will be queued if multiple pathway design tasks are submitted. Note that optimal stoichiometry does not guarantee a viable pathway design, as optStoic solves a linear programming (LP) problem that maximizes theoretical yield while enforcing elemental and charge balance. This can sometimes result in unrealistic stoichiometries that do not generate feasible pathways. Users may also need to allow ATP hydrolysis to drive reactions with a positive Gibbs energy change, making the overall stoichiometry thermodynamically feasible. This adjustment can potentially lead to alternative biosynthetic pathways. The web platform supports pathway searches, generating up to 10 distinct pathways, each with up to 10 reactions, for any overall stoichiometry provided by optStoic or entered manually. For novel reactions identified during the search, users can view the top 10 enzyme candidates, provided the underlying reaction rule yields at least 10 candidates. Furthermore, ΔG (Gibbs free energy change) predictions are available for all novel reactions. As standalone tools, dGPredictor [38] evaluates up to 5 reactions simultaneously, while EnzRank [23] assesses a single substrate-enzyme pair at a time. Both typically complete their analyses in under 2 minutes and can be executed repeatedly. In contrast, pathway searches generally require several hours to complete.

**Table 2:** Comparison of existing biosynthesis planning tools.

Tool	Year	Website	Input	Thermodynamic feasibility check	Enzyme candidates	Balanced	Database
novoStoic2.0	2025	https://novostoic.platform.moleculemaker.org/home	Start & Target	Yes (dGPredictor)	Yes (EnzRank)	Yes	MetaNetX, KEGG, RHEA
RetroBioCat [17]	2021	https://retrobiocat.com	Target	No	Yes (via literature)	No	Expert defined rules
RetroPath2.0 [13]	2018	https://www.myexperiment.org/workflows/4987.html	Target	Yes (group contribution)	Yes (Selenzyme [33])	No	MetaNetX, BRENDA
BioNavi [34]	2022	http://biopathnavi.qmclab.com/	Target	No	Yes (Selenzyme [33])	No	MetaCyc, KEGG, MetaNetX
PathPred [35]	2010	http://www.genome.jp/tools/pathpred/	Start & Target	No	No	No	KEGG
XTMS [36]	2014	http://xtms.issb.genopole.fr/	Target	Yes (group contribution)	Yes	Yes	MetaCyc, KEGG
enviPath [37]	2016	https://envipath.org/	Target	No	No	No	Expert curated reactions

novoStoic2.0 is by no means inclusive of all tasks needed to instantiate a pathway design. The pathway will ultimately have to be ported in a production strain. The strain will have to be engineered so that no carbon flux leaks away from the desired pathway. The expression levels of genes and translation rates of proteins along the pathway will have to be finely tuned to limit metabolic burden, and inhibitory controls will have to be ameliorated. There is already a rich literature of tools aimed at addressing these challenges (e.g., optKnock [39], optForce [40], RBSCalculator [41], etc.). Moving beyond, pathway carbon and energy efficiency, additional design considerations are equally important. These may include predicting the toxicity of intermediates, safeguarding against protein misfolding/aggregation, the presence of high-affinity product exporters, etc. For all these tasks, several computational tools are available to provide estimates [42–45]. Finally, perhaps the most challenging step is the identification or redesign of enzymes with the desired substrate specificity and activity to carry out novel conversion steps. Whereas in some cases promiscuous enzymes can be found or adapted through directed evolution [12,46], rapid advances in enzyme design using ML tools promise to automate this step [47–49]. We envision that many of these aforementioned tools will be integrated into future versions of novoStoic.

We anticipate that ML techniques will likely revolutionize pathway design in the same way that they have changed the landscape in protein folding and enzyme design. For instance, leveraging Large Language Models (LLMs) on up-to-date literature information could immediately inform pathway designs. For example, for the identified pathway shown in Fig 7, novoStoic classified step 3 converting tyrosol to HXT as a novel step, but a recent article by Chen et. al [12] suggested that the desired novel activity has already been assigned an enzyme that was not present yet in the database. Therefore, LLMs offer the promise of automating data mining from the literature and moving beyond simple SMILES encodings for metabolites and reactions. novoStoic2.0 already leverages some of these ML developments and offers a versatile platform to integrate additional ones in the future.

## Supporting information

S1 File Case study: L-tyrosine to hydroxytyrosol pathway design using novoStoic2.0. (DOCX)

S2 File Enzyme candidates for novel reaction identified in hydroxytyrosol synthesis. (CSV)

S3 File EnzRank scores for novel reaction identified in hydroxytyrosol synthesis. (CSV)

S4 File More examples of pathway design using novoStoic2.0. (DOCX)
