# Supplementary material for: novoStoic2.0: An integrated framework for pathway synthesis, thermodynamic evaluation, and enzyme selection
Source: PLoS Comput Biol. 2025 Aug 6;21(8):e1012516. doi: 10.1371/journal.pcbi.1012516 (PMC12338773; doi:10.1371/journal.pcbi.1012516)
Supplement: S1 File — (DOCX) [file pcbi.1012516.s001.docx]

**SUPPLEMENTARY INFORMATION**

**novoStoic2.0: An integrated framework for pathway synthesis, thermodynamic evaluation, and enzyme selection**

Vikas Upadhyay, Mohit Anand, Costas D. Maranas*

*Department of Chemical Engineering, The Pennsylvania State University, University Park, PA, 16802*

** Correspondence to be addressed to:* [*costas@psu.edu*](mailto:costas@psu.edu)

**Individual steps on the MMLI (Molecular Maker Lab) website to reach the synthesis planning of Hydroxytyrosol provided in the paper**

Table 1: Results obtained for overall stoichiometry when L-tyrosine and Hydroxytyrosol are chosen as primary precursor and target molecule. Solution (17) is chosen as input for pathway design


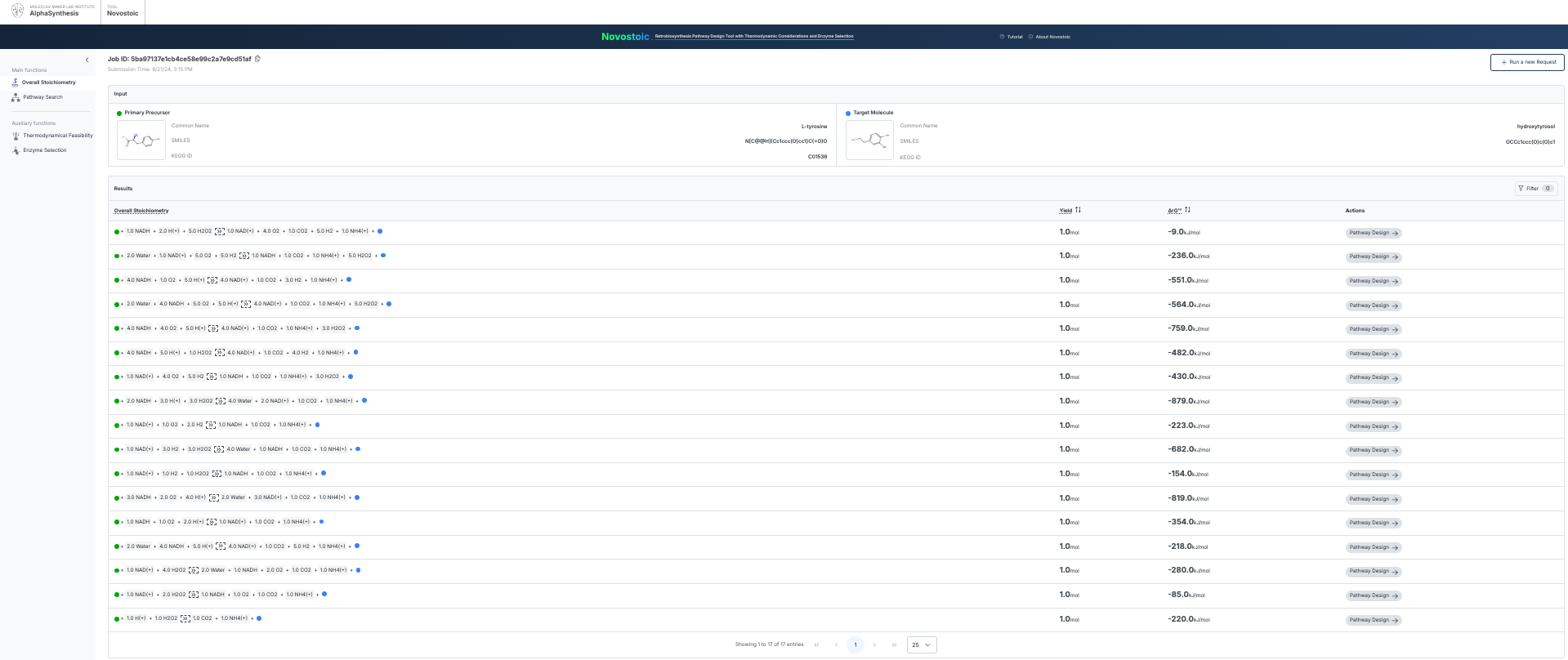


**Fig 1. optStoic results when L-Tyrosine(MNXM76) and Hydroxytyrosol(MNXM56953) are inputs for primary precursor and starting molecules respectively.** A list of 17 solutions were found for overall stoichiometry, i.e. when optStoic is run. In each solution, primary precursor is shown as green circle, and target molecules is shown as a blue circle. The rest of the molecules are co-reactants and co-products used to balance the overall reaction. The three other data points shown for each solution are yield of the target molecule, $\Delta_{r}G^{'o}$(estimation of standard Gibbs energy change) of the overall reaction, and actions for moving forward with pathway using the overall stoichiometry. $\Delta_{r}G^{'o}$ is estimated by dGPredictor and mean value of the estimation is shown.

| Index | Mean dG value | Overall Stoichiometry |
| --- | --- | --- |
| (1) | -9 kJ/mol | 1 NADH + 2 H^+^ + 5 H_2_O_2_ + 1 L-tyrosine ⬄ 1 NAD^+^ + 4 O_2_ + 1 CO_2_ + 5 H_2_ + 1 NH_4_^+^ + 1 hydroxytyrosol |
| (2) | -236 kJ/mol | 2 H_2_O + 1 NAD^+^ + 5 O_2_ + 5 H_2_ + 1 L-tyrosine ⬄ 1 NADH + 1 CO_2_ + 1 NH_4_^+^ 5 H_2_O_2_ + 1 hydroxytyrosol |
| (3) | -551 kJ/mol | 4 NADH + 1 O2 + 5 H^+^ + 1 L-tyrosine ⬄ 4 NAD^+^ + 1 CO_2_ + 3.0 H_2_ + 1.0 NH_4_^+^ + 1 hydroxytyrosol |
| (4) | -564 kJ/mol | 2 H_2_O + 4 NADH + 5 O_2_ + 5 H^+^ + 1 L-tyrosine ⬄ 4 NAD^+^ + 1 CO_2_ + 1 NH_4_^+^ + 5 H_2_O_2_ + 1 hydroxytyrosol |
| (5) | -759 kJ/mol | 4 NADH + 4 O_2_ + 5 H^+^ + 1 L-tyrosine ⬄ 4 NAD^+^ + 1 CO_2_ + 1.0 NH_4_^+^ + 3 H_2_O_2_ + 1 hydroxytyrosol |
| (6) | -482 kJ/mol | 4 NADH + 5 H^+^ + 1 H_2_O_2_ + 1 L-tyrosine ⬄ 4 NAD^+^ + 1 CO_2_ + 4 H_2_ + 1 NH_4_^+^ + 1 hydroxytyrosol |
| (7) | -430 kJ/mol | 1 NAD^+^ + 4 O_2_ + 5 H_2_ + 1.0 L-tyrosine ⬄ 1 NADH + 1 CO_2_ + 1 NH_4_^+^ + 3 H_2_O_2_ + 1 hydroxytyrosol |
| (8) | -879 kJ/mol | 2 NADH + 3 H^+^ + 3 H_2_O_2_ + 1 L-tyrosine ⬄ 4 H_2_O + 2 NAD^+^ + 1 CO_2_ + 1.0 NH_4_^+^ + 1 hydroxytyrosol |
| (9) | -223 kJ/mol | 1 NAD^+^ + 1 O_2_ + 2 H_2_ + 1 L-tyrosine ⬄ 1 NADH + 1 CO_2_ + 1.0 NH_4_^+^ + 1 hydroxytyrosol |
| (10) | -682 kJ/mol | 1 NAD^+^ + 3 H_2_ + 3 H_2_O_2_ + 1 L-tyrosine ⬄ 4 H_2_O + 1 NADH + 1 CO_2_ + 1 NH_4_^+^ + 1 hydroxytyrosol |
| (11) | -154 kJ/mol | 1 NAD^+^ + 1 H_2_ + 1 H_2_O_2_ + 1 L-tyrosine ⬄ 1 NADH + 1 CO_2_ + 1 NH_4_^+^ + 1 hydroxytyrosol |
| (12) | -819 kJ/mol | 3 NADH + 2 O_2_ + 4 H^+^ + 1 L-tyrosine ⬄ 2 H_2_O + 3 NAD^+^ + 1 CO_2_ + 1 NH_4_^+^ + 1 hydroxytyrosol |
| (13) | -354 kJ/mol | 1 NADH + 1 O_2_ + 2 H^+^ + 1.0 L-tyrosine ⬄ 1.0 NAD^+^ + 1 CO_2_ + 1 NH_4_^+^ + 1 hydroxytyrosol |
| (14) | -218 kJ/mol | 2 H_2_O + 4 NADH + 5 H^+^ + 1.0 L-tyrosine ⬄ 4.0 NAD^+^ + 1 CO_2_ + 5 H2 + 1 NH_4_^+^ + 1 hydroxytyrosol |
| (15) | -280 kJ/mol | 1 NAD^+^ + 4 H_2_O_2_ + 1.0 L-tyrosine ⬄ 2 H_2_O + 1 NADH + 2 O_2_ + 1 CO_2_ + 1 NH_4_^+^ + 1 hydroxytyrosol |
| (16) | -85 kJ/mol | 1 NAD^+^ + 2 H_2_O_2_ + 1 L-tyrosine ⬄1 NADH + 1.0 O_2_ + 1.0 CO_2_ + 1 NH_4_^+^ + 1 hydroxytyrosol |
| (17) | -220 kJ/mol | 1 H^+^ + 1.0 H_2_O_2_ + 1 L-tyrosine ⬄ 1.0 CO_2_ + 1.0 NH_4_^+^ + 1 hydroxytyrosol |


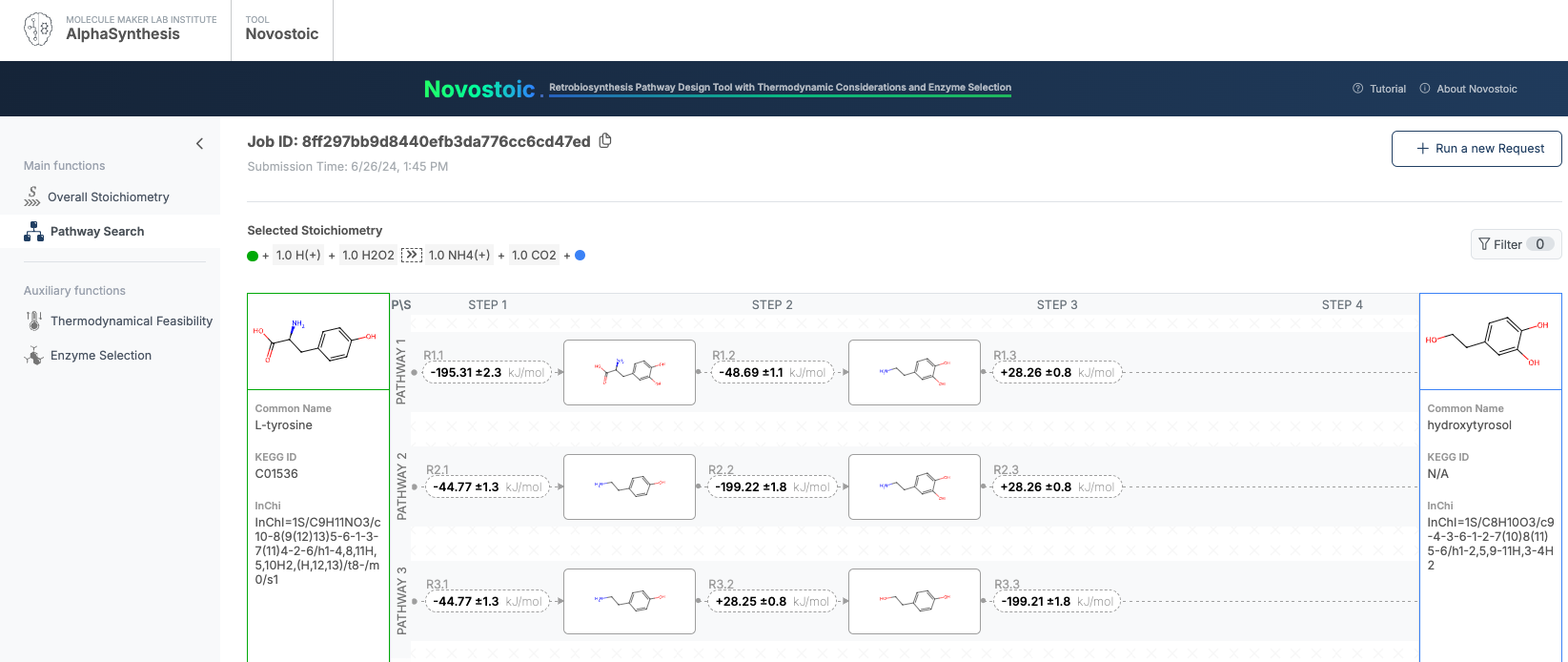


**Fig 2. Three 3-step pathways identified by the novoStoic using the highlighted stoichiometry in Table 1 (17).** novoStoic uses MILP formulation to utilize reaction rules that can connect the source to a target molecule based on the given stoichiometry. However, a single MILP solution can lead to multiple pathways depending on the order of application of reaction rules on the source and subsequence intermediate molecules. Here, we show three pathways using the same solution but changing the sequence of reaction rule application on the starting substrate based on the flux value of each reaction rule. The reaction rules for the identified pathways are MNXR102311 (1.0), MNXR114039 (1.0), and MNXR151676 (-1.0, meaning reverse directionality of the reaction).


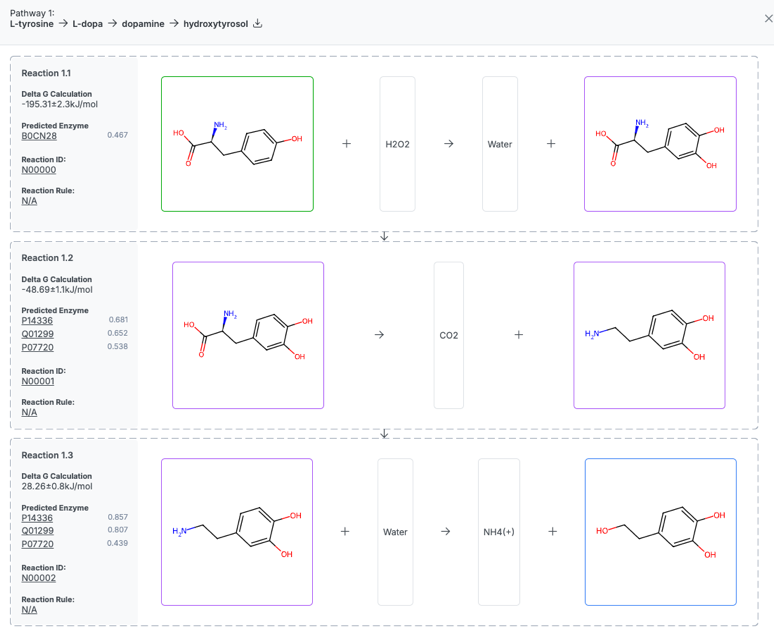


**Fig 3. Detailed visualization of the designed pathway 1 for L-tyrosine to hydroxytyrosol.** The web interface allows users to look for the individual reaction steps in detail along with the Standard Gibbs energy change estimated by dGPredictor and the rank-ordered known enzyme candidates for any novel reactions using EnzRank as well as enzyme information for the known reactions in the pathways, by clicking over the pathway. Here, the primary metabolites in each step of Pathway 1 are L-tyrosine, L-dopa, dopamine, and hydroxytyrosol respectively. Co-reactants and co-products of every reaction are also shown here. The first reaction (R1.1) is based on the reaction rule MNXR114039 (from MetaNetX database) is a peroxygenase reaction, the second reaction (R1.2) is based on the reaction rule MNXR151676 (from MetaNetX database) is a decarboxylation reaction and the third reaction (R1.3) is based on the reaction rule MNXR102311 (from MetaNetX database) is a transferase reaction where ammonia is displaced by a hydroxyl group.


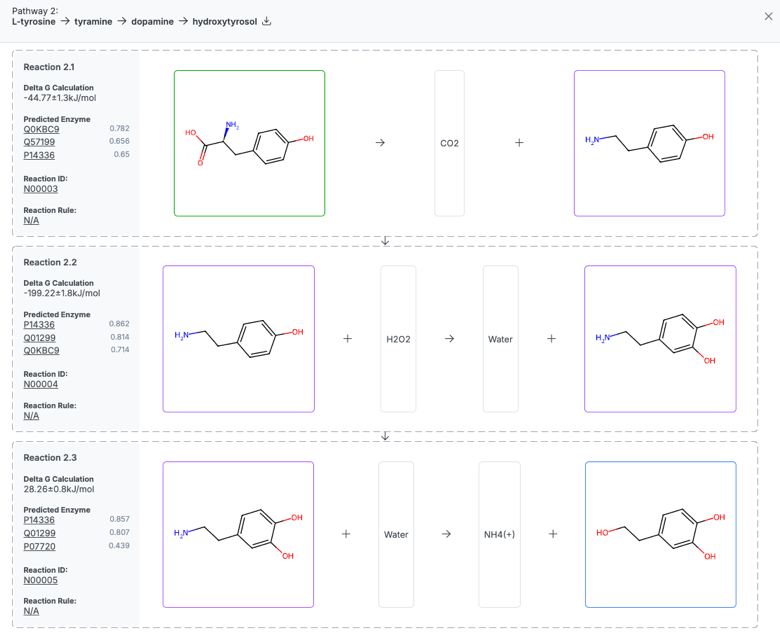


**Fig. 4. Detailed visualization of designed pathway 2 for L-tyrosine to hydroxytyrosol.** The primary metabolites in each step are L-tyrosine, tyramine, dopamine, and hydroxytyrosol, respectively (shown on top). Co-reactants and co-products of each reaction are also displayed. The first reaction (R2.1), based on the reaction rule MNXR151676 (from the MetaNetX database), is a decarboxylation reaction. The second reaction (R2.2), based on the reaction rule MNXR114039, is a peroxygenase reaction. The third reaction (R2.3), based on the reaction rule MNXR102311, is a transferase reaction where ammonia is displaced by a hydroxyl group.


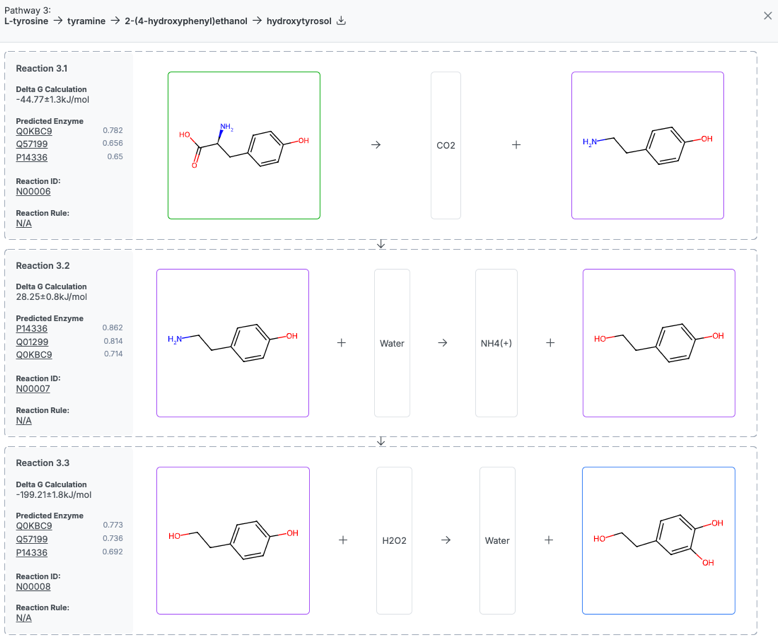


**Fig. 5. Detailed visualization of designed pathway 3 for L-tyrosine to hydroxytyrosol.** The primary metabolites in each step are L-tyrosine, tyramine, dopamine, and hydroxytyrosol, respectively. Co-reactants and co-products of each reaction are also shown. The first reaction (R2.1), based on the reaction rule MNXR151676 (from the MetaNetX database), is a decarboxylation reaction. The second reaction (R2.3), based on the reaction rule MNXR102311, is a transferase reaction where ammonia is displaced by a hydroxyl group. The third reaction (R2.2), based on the reaction rule MNXR114039, is a peroxygenase reaction.

**Visualization of novoStoic web-interface output for known stoichiometry exploring shorter pathways**

2 NADH + 2 O_2_ + 3 H^+^ + 1 L-tyrosine ⬄ 2 NAD^+^ + 1 CO_2_ + 1 NH_4_^+^ + 1 H_2_O_2_ + 1 hydroxytyrosol


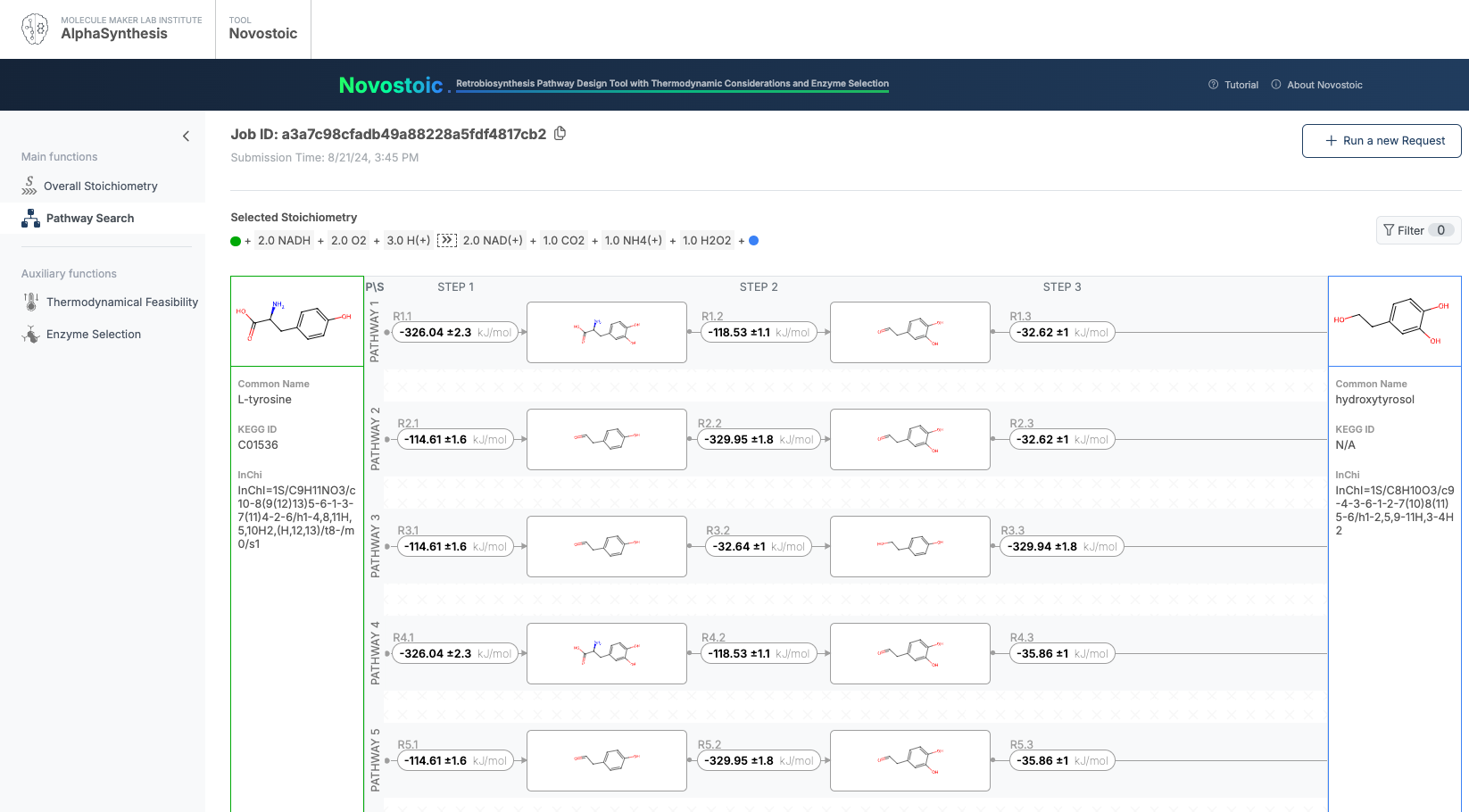

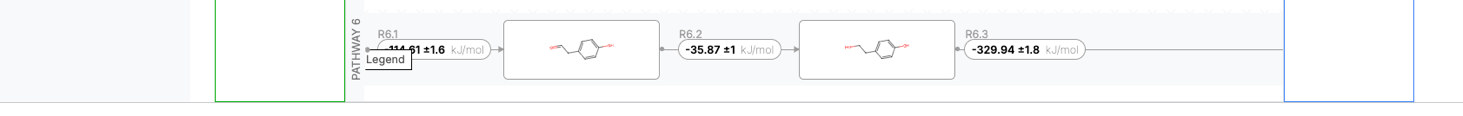


**Fig 6. Visualization of six 3-step pathways identified by the pathway search tool using the known stoichiometry.** The first solution from the MILP formulation provided three reaction rules and their corresponding fluxes: MNXR121797 (-1.0), MNXR100947 (1.0), and MNXR103537 (1.0). Pathways 1-3 were generated using these three reaction rules and fluxes. The second solution from the MILP formulation yielded three different reaction rules and fluxes: MNXR193435 (1.0), MNXR102281 (-1.0), and MNXR121802 (1.0). Pathways 4-6 were created using this second set of solutions. The reaction rules MNXR121797 and MNXR193435 are complementary, operating in opposite directions, as are MNXR100947 and MNXR121802. When flux values are also complementary, these complementary rules result in identical reaction steps. Therefore, despite differences in the reaction rules, R1.1 is functionally equivalent to R4.1, and R2.1 is equivalent to R5.1. The primary distinction lies in the cofactor usage: MNXR121802 uses NADH, whereas MNXR103537 utilizes NADPH.

Six 3-step pathways found by the pathway search tool using the known stoichiometry. These reaction rules are reordered using the visualization tool to figure out the order of reaction steps. First solution from the MILP formulation gave three reaction rules and their fluxes: MNXR121797 (-1.0), MNXR100947 (1.0), MNXR103537 (1.0). Pathways 1-3 are created using these three reaction rules and fluxes. Second solution from the MILP formulation gave three reaction rules and fluxes: MNXR193435 (1.0), MNXR102281 (-1.0), MNXR121802 (1.0). Pathways 4-6 are created using this second solution. The reaction rules MNXR121797 and MNXR193435 are complementary, meaning they operate in opposite directions. Similarly, MNXR100947 and MNXR121802 are also complementary. When the flux values are also complementary, these complementary rules yield identical reaction steps. Consequently, despite differences in the reaction rules, R1.1 is functionally equivalent to R4.1, and R2.1 is equivalent to R5.1. The primary distinction is that MNXR121802 uses NADH, whereas MNXR103537 utilizes NADPH.


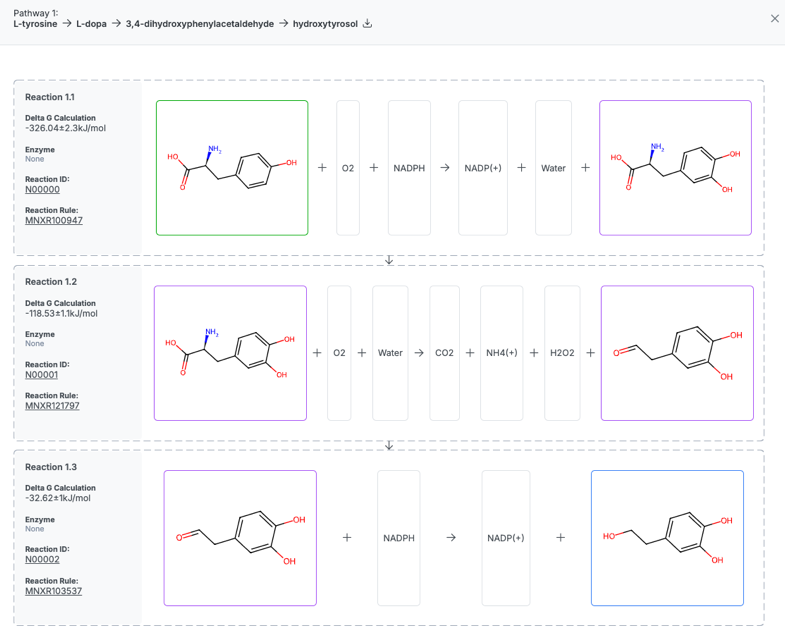


**Fig. 6. Detailed visualization of designed pathway 1 for L-tyrosine to hydroxytyrosol using second stoichiometry**. The primary metabolites in each step are L-tyrosine, L-dopa, 3,4-dihydroxyphenylacetaldehyde, and hydroxytyrosol, respectively (shown on top). Co-reactants and co-products of each reaction are also displayed. The first reaction (R1.1), based on the reaction rule MNXR100947 (from the MetaNetX database), is a monooxygenase reaction. The second reaction (R1.2), based on the reaction rule MNXR121797, simultaneously removes ammonia and reduces the carboxyl group to an aldehyde. The third reaction (R1.3), based on the reaction rule MNXR103537, is an oxidoreductase reaction.


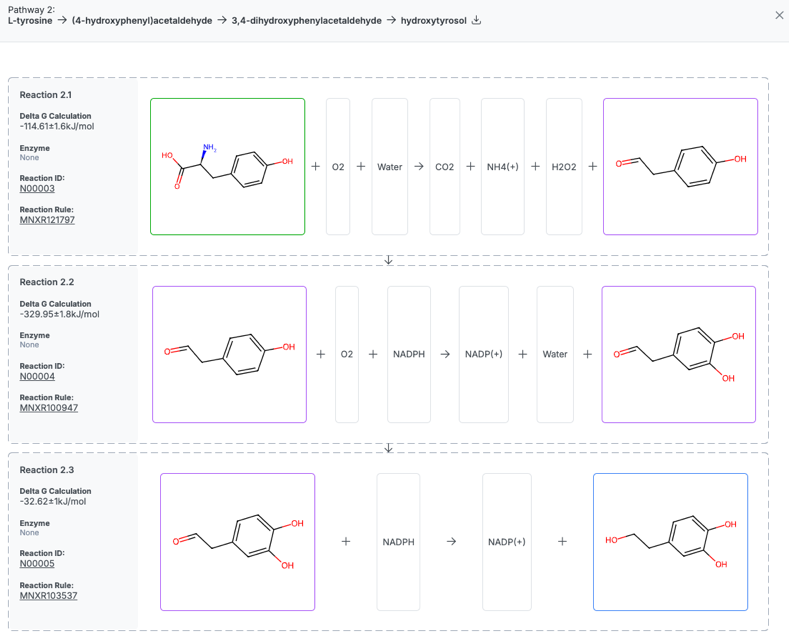


**Fig. 7. Detailed visualization of designed pathway 2 for L-tyrosine to hydroxytyrosol using second stoichiometry**. The primary metabolites in each step are L-tyrosine, 4-hydroxyphenylacetaldehyde, 3,4-dihydroxyphenylacetaldehyde, and hydroxytyrosol, respectively. Co-reactants and co-products of each reaction are also displayed. The first reaction (R3.1), based on the reaction rule MNXR121797 (from the MetaNetX database), simultaneously removes ammonia and reduces the carboxyl group to an aldehyde. The second reaction (R3.2), based on the reaction rule MNXR103537, is an oxidoreductase reaction. The third reaction (R3.3), based on the reaction rule MNXR100947, is a monooxygenase reaction.


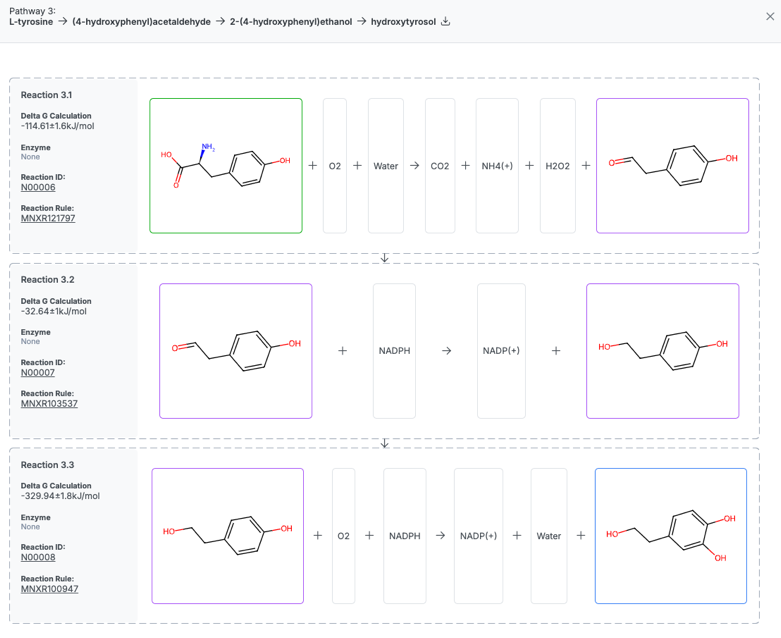


**Fig. 8. Detailed visualization of designed pathway 3 for L-tyrosine to hydroxytyrosol using second stoichiometry**. The primary metabolites in each step are L-tyrosine, 4-hydroxyphenylacetaldehyde, 2-(4-hydroxyphenyl)ethanol, and hydroxytyrosol, respectively. Co-reactants and co-products of each reaction are also displayed. The first reaction (R3.1), based on the reaction rule MNXR121797 (from the MetaNetX database), simultaneously removes ammonia and reduces the carboxyl group to an aldehyde. The second reaction (R3.2), based on the reaction rule MNXR100947, is a monooxygenase reaction. The third reaction (R3.3), based on the reaction rule MNXR103537, is an oxidoreductase reaction.
