# Supplementary material for: novoStoic2.0: An integrated framework for pathway synthesis, thermodynamic evaluation, and enzyme selection
Source: PLoS Comput Biol. 2025 Aug 6;21(8):e1012516. doi: 10.1371/journal.pcbi.1012516 (PMC12338773; doi:10.1371/journal.pcbi.1012516)
Supplement: S4 File — (DOCX) [file pcbi.1012516.s004.docx]

**SUPPLEMENTARY INFORMATION**

**novoStoic2.0: An integrated framework for pathway synthesis, thermodynamic evaluation, and enzyme selection**

Vikas Upadhyay, Mohit Anand, Costas D. Maranas*

*Department of Chemical Engineering, The Pennsylvania State University, University Park, PA, 16802*

** Correspondence to be addressed to:* [*costas@psu.edu*](mailto:costas@psu.edu)

**Pyruvate to Ethanol**


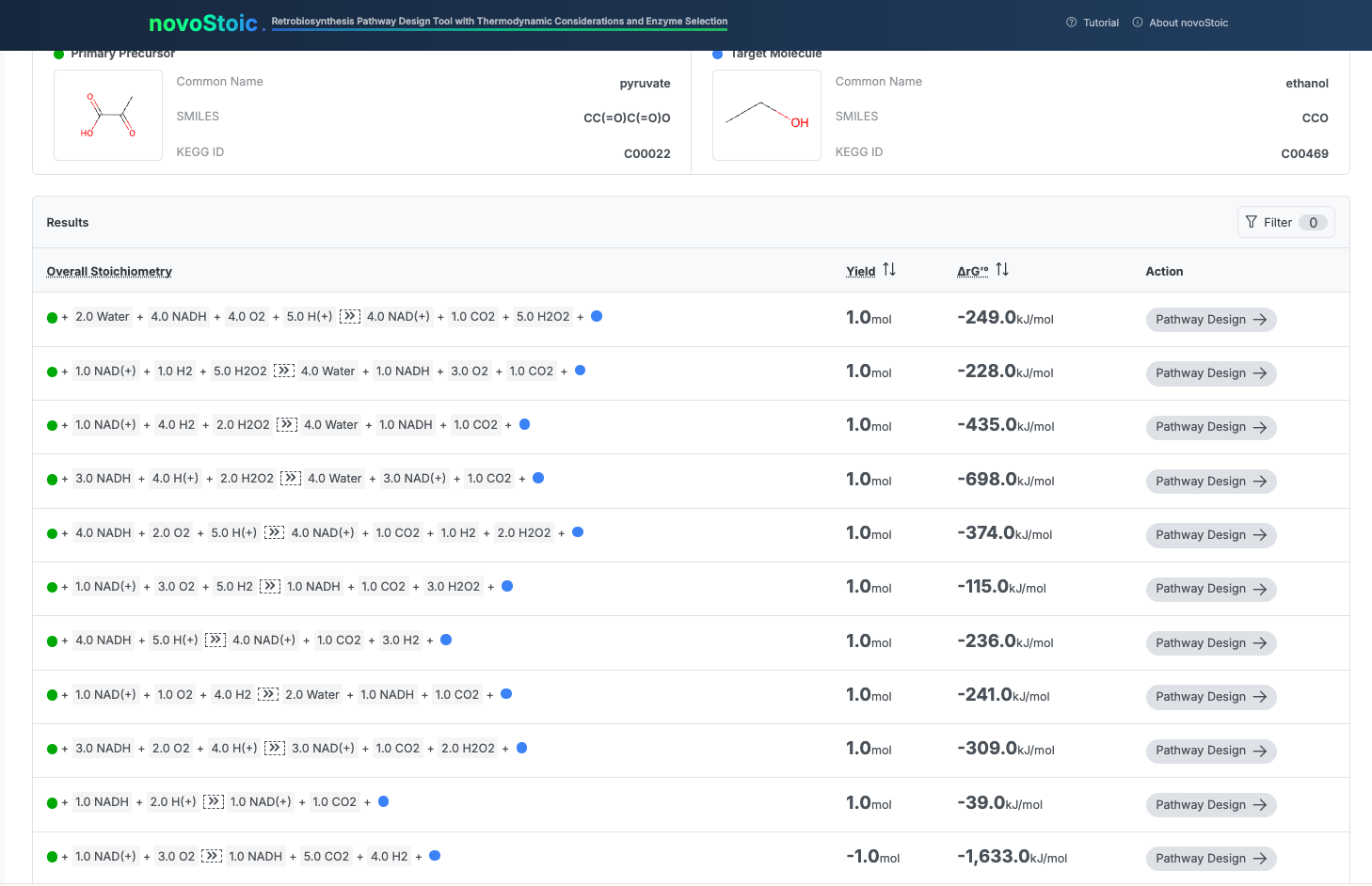


**Fig 1.** **optStoic results, when Pyruvate (MNXM23) and Ethanol (MNXM1108092) are inputs for primary precursor and starting molecules respectively.** A list of 11 solutions were found for overall stoichiometry, i.e. when optStoic is run. In each solution, primary precursor is shown as green circle, and target molecules is shown as a blue circle. The rest of the molecules are co-reactants and co-products used to balance the overall reaction. The three other data points shown for each solution are yield of the target molecule, $\Delta_{r}G^{'o}$(estimation of standard Gibbs energy change) of the overall reaction, and actions for moving forward with pathway using the overall stoichiometry. $\Delta_{r}G^{'o}$ is estimated by dGPredictor and mean value of the estimation is shown.

| **Index** | **Mean dG value** | **Overall Stoichiometry** |
| --- | --- | --- |
| (1) | -249 kJ/mol | 2 H_2_O + 4 NADH + 4 O_2_ + 5 H^+^ + 1 pyruvate ⬄ 4 NAD^+^ + 1 CO_2_ + 5 H_2_O_2_ + 1 ethanol |
| (2) | -228 kJ/mol | 1 NAD^+^ + 5 H_2_O_2_ + 1 H_2_ + 1 pyruvate ⬄ 4 H_2_O +1 NADH + 3 O_2_ + 1 CO_2_ + 1 ethanol |
| (3) | -435 kJ/mol | 1 NAD^+^ + 4 H_2_ + 2 H_2_O_2_ + 1 pyruvate ⬄ 4 H_2_O + 1 NADH + 1 CO_2_ + 1 ethanol |
| (4) | -698 kJ/mol | 3 NADH + 4 H^+^ + 2 H_2_O_2_ + 1 pyruvate ⬄ 4 H_2_O + 3 NAD^+^ + 1 CO_2_ + 1 ethanol |
| (5) | -374 kJ/mol | 4 NADH + 2 O_2_ + 5 H^+^ + 1 pyruvate ⬄ 4 NAD^+^ + 1 CO_2_ + 1 H_2_ + 2 H_2_O_2_ + 1 ethanol |
| (6) | -115 kJ/mol | 1 NAD^+^ + 3 O_2_ + 5 H_2_ + 1 pyruvate ⬄ 1 NADH + 1 CO_2_ + 3 H_2_O_2_ + 1 ethanol |
| (7) | -236 kJ/mol | 4 NADH + 5 H^+^ + 1 pyruvate ⬄ 4 NAD^+^ + 1 CO_2_ + 3 H_2_ + 1 ethanol |
| (8) | -241 kJ/mol | 1 NAD^+^ + 1 O_2_ + 4 H_2_ + 1 pyruvate ⬄ 2 H_2_O + 1 NADH + 1 CO_2_ + 1 ethanol |
| (9) | -309 kJ/mol | 3 NADH + 2 O_2_ + 4 H^+^ + 1 pyruvate ⬄ 3 NAD^+^ + 1 CO_2_ + 2 H_2_O_2_ + 1 ethanol |
| (10) | -39 kJ/mol | 1 NADH + 2 H^+^ + 1 pyruvate ⬄ 1 NADH + 1 CO_2_ + 1 ethanol |
| (11) | -1633 kJ/mol | 1 NADH + 3 O_2_ + 1 pyruvate ⬄ 1 NADH + 5 CO_2_ + 4 H_2_ + 1 ethanol |

**Table 1.** Results obtained for overall stoichiometry when Pyruvate and Ethanol are chosen as primary precursor and target molecule. Solution (10) is chosen as input for pathway design

**Pyruvate Ethanol**

**Overall stoichiometry (10):** $C_{3}H_{3}O_{3}+NADH+2 H^{+} \to C_{2}H_{6}O+NAD^{+}+CO_{2}$


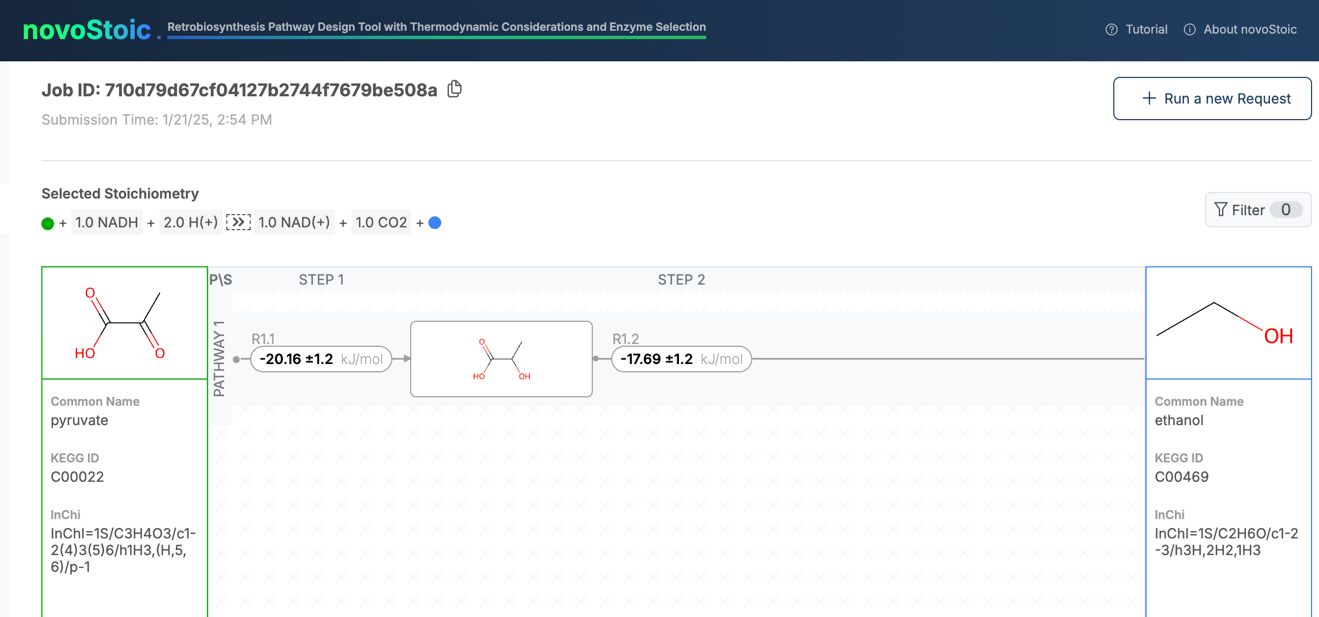


**Fig 2. One 2-step pathways identified by the novoStoic using the highlighted stoichiometry in Table 1 (10).** novoStoic uses MILP formulation to utilize reaction rules that can connect the source to a target molecule based on the given stoichiometry. Here, we show one pathway using the same solution. The reaction rules for the identified pathways are MNXR100326 (-1.0) and MNXR114039 (-1.0) (-1.0, meaning reverse directionality of the reaction)


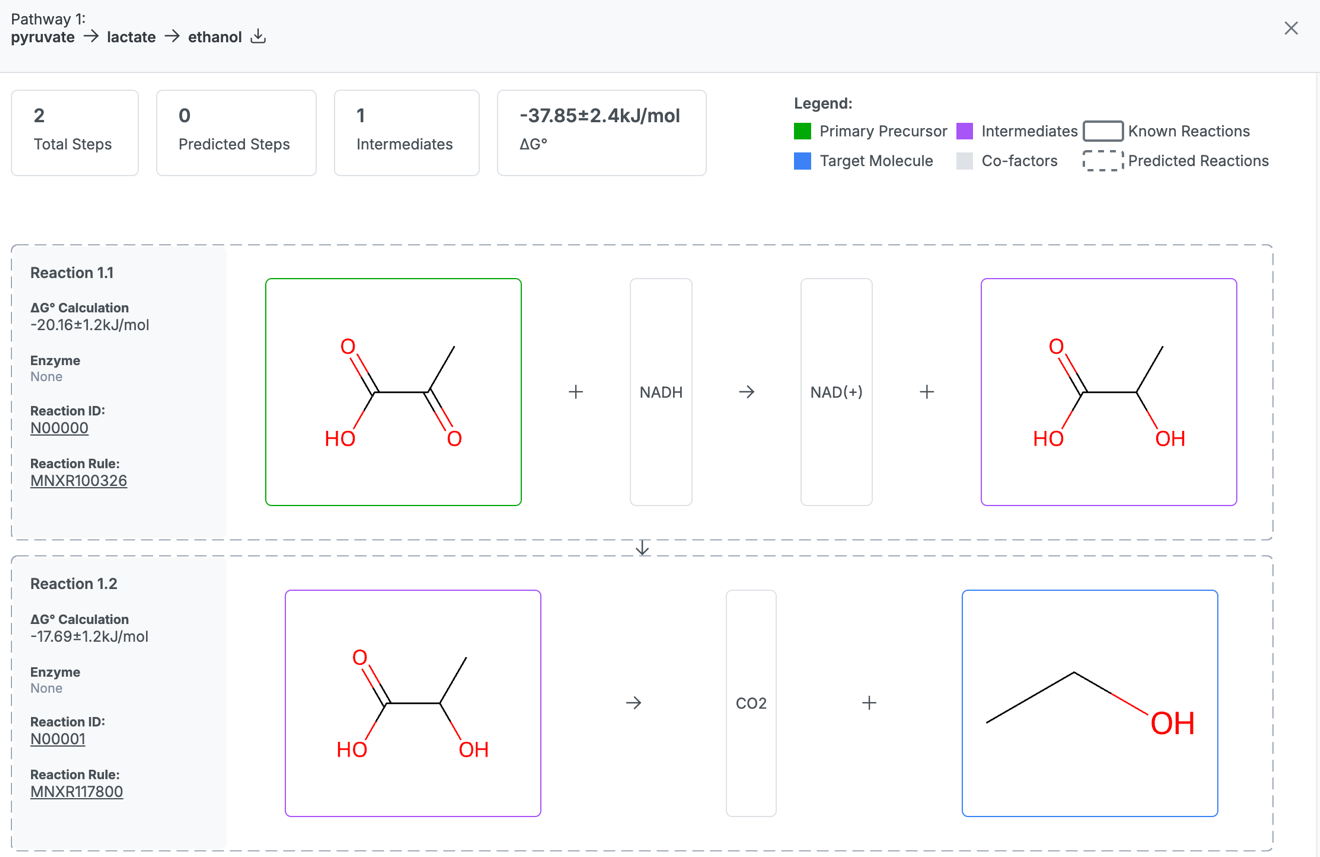


**Fig 3. Detailed visualization of the designed pathway.** The web interface allows users to look for the individual reaction steps in detail along with the Standard Gibbs energy change estimated by dGPredictor and the rank-ordered known enzyme candidates for any novel reactions using EnzRank as well as enzyme information for the known reactions in the pathways, by clicking over the pathway. Here, the primary metabolites in each step of Pathway 1 are pyruvate, lactate, and ethanol respectively. Co-reactants and co-products of every reaction are also shown here. The first reaction (R1.1) is based on the reaction rule MNXR100326 (from MetaNetX database) is a dehydrogenase reaction, the second reaction (R1.2) is based on the reaction rule MNXR117800 (from MetaNetX database) is a decarboxylation reaction.

**Fig 4. A 2-step pathway from pyruvate to ethanol the given overall stoichiometry #10 from Table 1 based on existing reactions.** Step 1, R1, of the pathway is a glycerol dehydrogenase reaction, based on the reaction rule extracted from the MetaNetX reaction MNXR100326. Step 2, R2, of the pathway is a decarboxylase reaction, based on the reaction rule extracted from the MetaNetX reaction MNXR117800.

**3 methyl-2-oxobutanoate (Ketoisovalerate) to isobutanol**


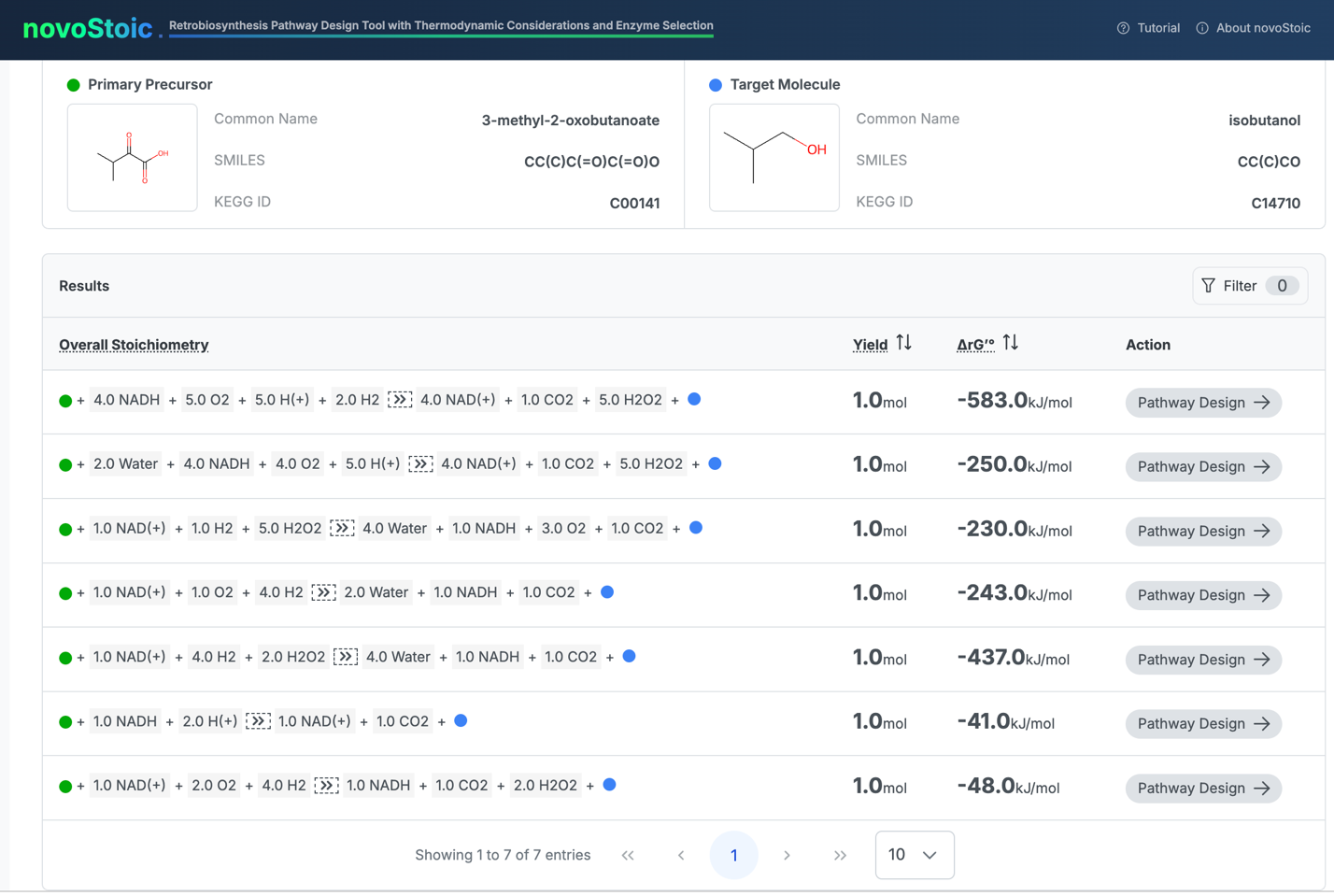


**Fig 5.** **optStoic results, when Ketoisovalerate (MNXM732866) and Isobutanol (MNXM5188) are inputs for primary precursor and starting molecules respectively.** A list of 7 solutions were found for overall stoichiometry, i.e. when optStoic is run. In each solution, primary precursor is shown as green circle, and target molecules is shown as a blue circle. The rest of the molecules are co-reactants and co-products used to balance the overall reaction. The three other data points shown for each solution are yield of the target molecule, $\Delta_{r}G^{'o}$(estimation of standard Gibbs energy change) of the overall reaction, and actions for moving forward with pathway using the overall stoichiometry. $\Delta_{r}G^{'o}$ is estimated by dGPredictor and mean value of the estimation is shown.

| **Index** | **Mean dG value** | **Overall Stoichiometry** |
| --- | --- | --- |
| (1) | -583 kJ/mol | 4 NADH + 5 O_2_ + 5 H^+^ +2 H_2_ + 1 pyruvate ⬄ 4 NAD^+^ + 1 CO_2_ + 5 H_2_O_2_ + 1 ethanol |
| (2) | -250 kJ/mol | 2 H_2_O + 4 NADH + 4 O_2_ + 5 H^+^ + 1 pyruvate ⬄ 4 NAD^+^ +1 CO_2_ + 5 H_2_O_2_ + 1 ethanol |
| (3) | -230 kJ/mol | 1 NAD^+^ + 1 H_2_ + 5 H_2_O_2_ + 1 pyruvate ⬄ 4 H_2_O + 1 NADH + 3 O_2_ + 1 CO_2_ + 1 ethanol |
| (4) | -243 kJ/mol | 1 NAD^+^ + 1 O_2_ + 4 H_2_ + 1 pyruvate ⬄ 2 H_2_O + 1 NADH + 1 CO_2_ + 1 ethanol |
| (5) | -437 kJ/mol | 1 NAD^+^ + 4 H_2_ + 2 H_2_O_2_ + 1 pyruvate ⬄ 4 H_2_O + 1 NADH + 1 CO_2_ + 1 ethanol |
| (6) | -41 kJ/mol | 1 NADH + 2 H^+^ + 1 pyruvate ⬄ 1 NAD^+^ + 1 CO_2_ + 1 ethanol |
| (7) | -48 kJ/mol | 1 NAD^+^ + 2 O_2_ + 4 H_2_ + 1 pyruvate ⬄ 1 NADH + 1 CO_2_ + 2 H_2_O_2_ + 1 ethanol |

**Table 2.** Results obtained for overall stoichiometry when Ketoisovalerate and Isobutanol are chosen as primary precursor and target molecule. Solution (6) is chosen as input for pathway design

**Ketoisovalerate IBA**

**Overall stoichiometry (6):** $C_{5}H_{7}O_{3}+NADH\to C_{4}H_{10}O + NAD^{+}+CO_{2}$

**Fig 6. A 2-step pathway from Ketoisovalerate to Isobutanol (IBA) for the given overall stoichiometry #6 from Table 2 based on existing reactions.** Step 1, R1, of the pathway is a decarboxylase reaction, based on the reaction rule extracted from the MetaNetX reaction MNXR117800. Step 2, R2, of the pathway is a glycerol dehydrogenase reaction, based on the reaction rule extracted from the MetaNetX reaction MNXR100326.
